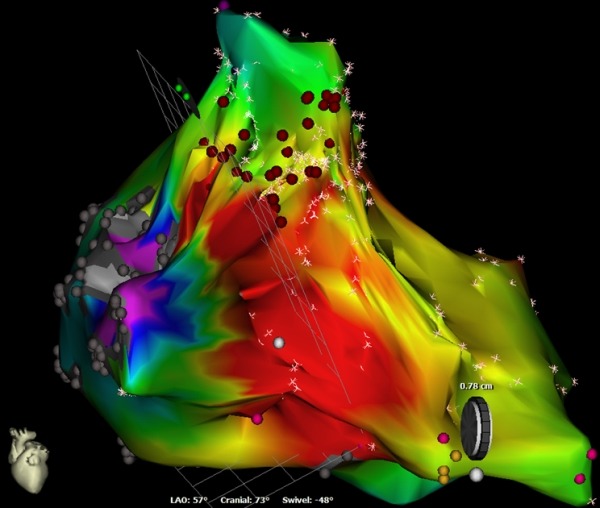# Idiopathic Right Atrial Scar

**DOI:** 10.1016/s0972-6292(16)30718-5

**Published:** 2014-01-01

**Authors:** Hygriv B Rao, AK Sivaprasad

**Affiliations:** KIMS Hospitals, Hyderabad 500003, India

**Keywords:** Idiopathic Right Atrial Scar

## Idiopathic Right Atrial Scar

**Figure 1:**
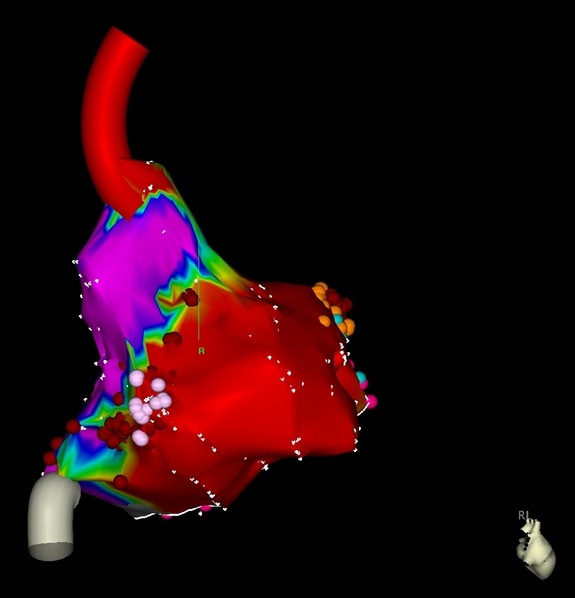


**Figure 2:**
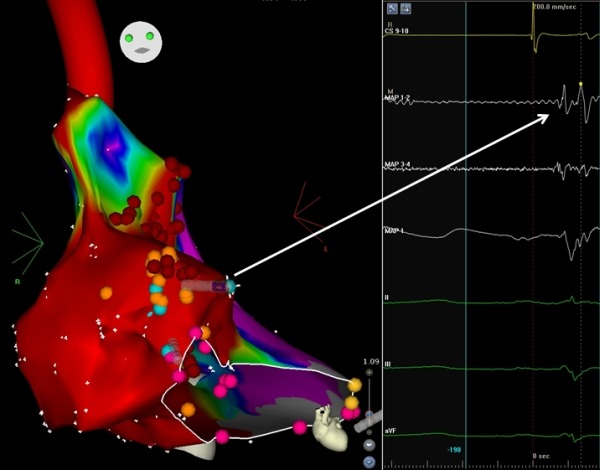


Right atrial scars participating in macro reentrant tachycardia circuits are generally seen in patients following cardiac surgery for congenital heart disease. Right and left atrial scars without any prior cardiac surgery have been reported but are rare [1,2]. A 65 year old farmer had recurrent drug refractory atrial tachycardia for which he underwent an EP study with three dimensional mapping and radiofrequency ablation. He was not a diabetic or hypertensive, had a structurally normal heart with preserved biventricular function, and normal coronaries. He had not undergone any cardiac surgery prior. Three dimensional mapping was done using CARTO 3 (Biosence, Webster). Sinus rhythm mapping of the right atrium (RA) showed a large atrial scar, defined as electrically silent areas with no recordable activity or bipolar voltage amplitude < 0.25 V. The scar was distributed over posterior, anterior and anterolateral areas near the tricuspid annulus (TA). Mapping during tachycardia showed macro reentrant tachycardia (cycle length =370 msec) propagating around RA scar. Figure 1 shows the sinus rhythm bipolar voltage map of the right atrium (RA) shows the large extent of the RA scar. Figure 2 shows the late split atrial potentials mapped at the scar borders. Figure 3 shows activation map of atrial tachycardia, demonstrating "Early meets late" at the upper scar border.

## Figures and Tables

**Figure 3:**